# Studies of Lipid Monolayers Prepared from Native and Model Plant Membranes in Their Interaction with Zearalenone and Its Mixture with Selenium Ions

**DOI:** 10.1007/s00232-017-9958-x

**Published:** 2017-04-27

**Authors:** Barbara Gzyl-Malcher, Maria Filek, Elżbieta Rudolphi-Skórska, Apolonia Sieprawska

**Affiliations:** 1 Faculty of Chemistry, Jagiellonian University, Ingardena 3, 30-060 Kraków, Poland; 2 Polish Academy of Science, The Franciszek Górski Institute of Plant Physiology, Niezapominajek 21, 30-239 Kraków, Poland; 3 Institute of Biology, Pedagogical University, Podchorążych 3, Kraków, Poland

**Keywords:** Langmuir monolayer, Lipid, Selenium, Wheat calli, Zearalenone

## Abstract

The impact of zearalenone and selenate ions on the monolayers of 1,2-dipalmitoyl-phosphatidylcholine (DPPC), 1,2-dipalmitoyl-3-trimethylammonium-propane (DPTAP), and the lipid mixtures (phospholipids and galactolipids) extracted from wheat plasmalemma has been studied using Langmuir trough technique and Brewster angle microscopy (BAM). The zearalenone is a mycotoxin that exerts toxic effects on the cells of plants and animals. Monolayers’ properties were characterized by surface pressure (*π*)—molecular area (*A*) isotherms. It was found that zearalenone interacts with lipid monolayers causing their expansion. The selenate ions, added to the subphase together with zearalenone, reduce the effect of this mycotoxin on the surface properties of lipid films.

## Introduction

Monolayers, the most important model system in the investigations of the specific reactions between lipids, as well as the changes in their structure influenced by the adsorption of substances from solution, are also used for the description of the nature and the possibility of the interactions in/with plant membranes (Brezesinski and Mohwald 2003; Gzyl-Malcher et al. 2008; Rudolphi-Skórska and Sieprawska 2016; Stefaniu et al. 2014). On the basis of Langmuir monolayer technique, some steps in the mechanisms of physiological processes, especially those connected with the influence of hormones (Filek et al. 2005; Gzyl et al. 2004; Wnętrzak et al. 2013), organic and non-organic ions (Gzyl-Malcher et al. 2009, 2011; Rudolphi-Skórska et al. 2014) have been interrelated. One of the important problems in plant physiology is to protect the cells against the mycotoxin accumulation (Diamond et al. 2013). The changes in climate conditions favor the growth and development of fungi on plants. Most of the toxins absorbed in plant tissues, especially in those used as food, are harmful to both animal and human cells (Gilbert and Tekauz 2000; Gromadzka et al. 2008; Ngoko et al. 2008).

**Fig. 1:**
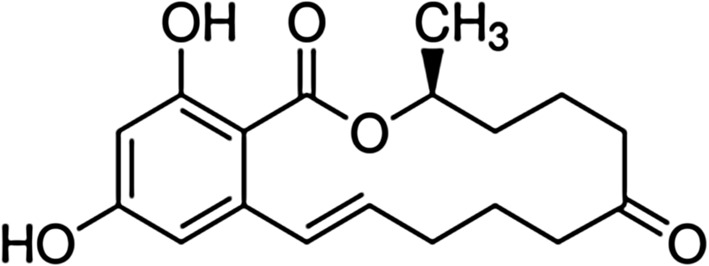
Chemical structure of zearalenone; (3*S*,11*E*)-14,16-dihydroxy-3-methyl-3,4,5,6,9,10-hexahydro-1*H*-2-benzoxacyclotetradecine-1,7(8*H*)-dione (C_18_H_22_O_5_)

Zearalenone (ZEA) (6-[10-hydroxy-6-oxo-trans-1-undecenyl]-B-resorcyclic acid lactone) with the molecular formula C18H22O5, also known as RAL and mycotoxin F2, is a non-ionic compound produced by genus *Fusarium* in cereals (Duca et al. 2006). ZEA is a natural form of the trans isomer and an S configuration with its methyl group in position C3 (Kuo et al. 1967) (Fig. 1). This mycotoxin is a non-steroidal substance, with the chemical structure similar to estrogen. The major part of ZEA molecule represents a hydrophobic region, but the presence of two hydroxyl groups makes this fragment capable to interact with polar substances—its solubility in water at 25 °C is found to be equal to 20 mg/dm^3^ (62.8 μM) (Gilbert and Şenyuva 2008). ZEA is better soluble in alkaline solutions, alcohols, acetone, benzene, and chloroform. When it is present in small doses in plants, it shows activity similar to plant hormones in some physiological processes (Biesaga-Kościelniak and Filek 2010; Filek et al. 2010). In higher concentrations, ZEA causes the most commercially devastating diseases of food crops, and as a result, world agriculture suffers massive produce loss each year (Kotowicz et al. 2014; Popovski and Celar 2013). Fusarium head blight is a recurrent disease of wheat and barley across the world, also including Europe (Bottalico and Perrone 2002). ZEA and its derivatives have multitude of effects on eukaryotic cells, among which, the most important seems to be the inhibition of protein synthesis (Pinton et al. 2012). However, the mechanisms of mycotoxin regulation leading to the accumulation of toxins in the plant, and consequently in human and animal cells, are still partially unknown. It is assumed that ZEA is bind to estrogen receptors in the membranes of animal’s cells that cause hyperestrogenism, resulting in various reproductive and infertility problems, because any compound with hormonal activity may act carcinogenically or genotoxically (Moss 2002; Kuciel-Lisieska et al. 2008). Therefore, an important issue is to eliminate the toxicity of zearalenone by the binding of its molecules by organic and inorganic substances (Crespo-Sempere et al. 2015; Chestnut et al. 1992; Gowda and Ledoux 2008; Salah-Abbès et al. 2009).

As the membranes are the structures involved in the regulation of many physiological processes, the aim of these studies was to elucidate the possibility of interaction of ZEA and selenate ions with lipid monolayers. From (Filek et al. 2002), it was established that ZEA, in spite of its non-steroid character, may be partly incorporated into the lipid layer and may modify the membrane structure. Selenate ions were used as a factor which can serve as a protector against ZEA toxic effects. It was indicated in many articles that selenium supplementation of soils not only improved the plant growth and development, but also served as the protector against the environmental stresses (Xue and Hartikainen 2000; Filek et al. 2009; Ibrahim 2014; Seppänen et al. 2003; Sieprawska et al. 2015). In these experiments, monolayers were built from lipids extracted from wheat cells. Calli cells in in vitro conditions are usually used to examine the direct interaction of cell membranes with organic and non-organic substances which can be added to the culture media (Tiemann and Dänicke 2007; Filek et al. 2009). Additionally, the relations of ZEA and selenate ions with the native lipids extracted from calli were compared with the effects of these substances on monolayers prepared from model lipids: DPPC (1,2-dipalmitoyl-phosphatidylcholine)—zwitterionic lipid, as an abundant phospholipid in plant cell membranes (Furt et al. 2011) and DPTAP (1,2-dipalmitoyl-3-trimethylammonium-propane)—positively charged lipid. DPTAP was used to mimic the presence of the cationic charge localized in the membrane as it is critical in understanding the electrostatic effects in the biological membranes (Ege et al., 2005, Gzyl-Malcher et al. 2009, Klausen et al. 2016) and/or the influence of cationic domain size on enzyme stability (Litt et al. 2009). The relationship between the phase behavior of cationic and modification of the monolayer structure in the presence of DPTAP was described by Ma et al. (2007) and Schmatko et al. (2014), respectively. In the future, we intend to continue working on the mixtures also containing negatively charged lipids. The estimation of the physicochemical parameters of monolayers, changed by the interaction with ZEA and ZEA + SeO_4_^2−^, will allow the interpretation of the effects of these substances on plant membranes. The parameter that can be determined directly from the isotherm surface pressure (*π*)—molecular area (*A*) is the limiting area, *A*_lim_ that is obtained by extrapolation of the steepest linear part of the isotherm at the end compression to zero pressure (Gaines 1966). The monolayer compressibility modulus, *C*_s_^−1^ = −(*dπ*/*d*ln*A*), (Davies and Rideal 1963) is often calculated because it provides information on the monolayer state (the more condensed monolayer, the higher value of *C*_s_^−1^).

## Materials and Methods

### Plant Material

Seeds of spring wheat cv. Raweta after germination in sterile Petri dishes filled with deionized water (2 days, 20 °C, dark), were placed into pots with a mixture soil:peat:sand (3:2:1; v/v/v) and cultured in greenhouse at 20/17 °C (day/night), and 16/8 h day/night photoperiod [400 µmol(quantum).m^−2^ s^−1^ light] till developing the first anthers. Immature embryos were chosen as material for in vitro cultures. After isolation from seeds, embryos were sterilized (70% ethanol and 10% Domestos), washed with distilled water, and then placed on Petri dishes containing Murashige and Skoog (MS) medium (Murashige and Skoog 1962) supplemented with 2 mg/ml 2,4-D. To receive undifferentiated cells of calli, tissues were cultured for 2 months at 25 °C. Every 3 weeks calli were transferred into fresh media.

Undifferentiated calli were transferred for 7 days into new MS media supplemented with: ZEN (30 µM), Na_2_SeO_4_ (15 µM), and the mixtures ZEN + Na_2_SeO_4_. ZEN concentration (stressogenic effect) was chosen in preliminary experiments (about 35% of damaged cells—on the basis of the cell viability). Selenate contents that act as potential protectors were selected according to earlier studies (Filek et al. 2009; Rudolphi-Skórska and Sieprawska 2016). MS media without additional supplementation served as a control.

For each treatment, 10 Petri dishes (at about 1 g of the fresh mass of calli) were used. All experiments were repeated 5 times.

### Biochemical Analysis

#### Lipid Extraction from Plasmalemma of Callus Cells

To obtain a calli plasmalemma, the method described earlier by Gzyl-Malcher et al. (Gzyl-Malcher et al. 2007) was used. Calli were homogenized in mixture containing 250 mM sucrose, 2.5 mM dithiothreitol (DTT), 1 mM phenylmethylsulfonyl fluoride (PMSF), 1 M ethylenediaminetetraacetic acid (EDTA), and 10 mM tris(hydroxymethyl)aminomethane (Tris) (pH = 7.5) at 4 °C. After two-step centrifugation at 10,000×*g* and at 8000×*g*, the microsomal fraction was re-suspended in 5 mM KP buffer (pH 7.8) with 250 mM sucrose and 5 mM KCl and added to a phase mixture consisting of 6.5% (w/w) dextran T500, 6.5%(w/w) polyethylene glycol (PEG4000), 250 mM sucrose, and 4 mM KCl in 5 mM KP buffer (pH 7.8). After a three-step phase partitioning, plasmalemma, included in the upper phase was centrifuged at 100,000×*g* with 250 mM sucrose and 1 mM ethylenebis(oxyethylenenitrilo)tetraacetic acid (EGTA) in 10 mM Tris buffer (pH 7.4).

For lipid extraction, obtained plasmalemma was extracted with a mixture of chloroform/isopropanol (1:1 v/v), and re-extracted with chloroform according to a modified method of Bligh and Dyer (Bligh and Dyer, 1959). The fractions of phospholipids (PL) and glycolipids (monogalactosyldiacylglycerol, MGDG and digalactosyldiacylglycerol, DGDG) were separated using adsorptive and distributive column chromatography on silica acid under nitrogen at low pressure and then purified by thin-layer chromatography. The quantitative and qualitative identification of individual phospholipids was established on the basis of visualization of TLC bands with adequate phospho- and galactolipid standards (Block et al. 1983).

Fatty acid composition in all the obtained lipid fractions was detected by gas chromatography (Hewlett Packard, USA), after reaction with 14% BF_3_ (in methanol). Chromatograph was equipped with capillary column (30 m × 0.25 mm) and heptadecanoic acid (17:0) was added as internal standard. Qualitative and quantitative analyses of fatty acids were made using appropriate standards.

#### Chemicals

Zearalenone and sodium selenate were purchased from Sigma-Aldrich Company (St, Louis, MO). 1,2-dipalmitoyl-phosphatidylcholine (DPPC) and 1,2-dipalmitoyl-3-trimethylammonium-propane (DPTAP) were obtained from Avanti Polar Lipids (Alabaster, AL). Chloroform (Merck, Germany) was the spreading solvent. The subphase was re-distilled water, purified by a Milli-Q system, with a specific resistance above 18.2 MQ cm^−1^.

#### Langmuir Monolayers

The experiments were performed using Langmuir trough (NIMA, Coventry, UK) of total surface area 300 cm^2^. Surface pressure (*π*) was detected with the accuracy of ±0.1 mN/m using a Wilhelmy plate made of filter paper (ashless Whatman Chr1) connected to an electrobalance. Monolayers were prepared by spreading chloroform solutions of lipids on the surface of water or ZEA/ZEA + Na_2_SeO_4_ water solutions. ZEA was added to the water subphase at 15 and 30 μM and selenate ions at 15 μM. In experiments with mixed lipid–zearalenone monolayers, the molar ratio of DPPC to zearalenone was kept constant (1:1). All molecules (DPPC, DPTAP, and ZEA) were used for the calculation of the area [Å^2^/molecule]. Premixed solutions of lipids with zearalenone in chloroform were spread on the surface of pure water or on the aqueous solution of selenate. All experiments were performed at 20 °C, with the rate of monolayer compression fixed as 5 Å^2^ molecule^−1^ min^−1^ and repeated three or four times to ensure high reproducibility of obtained isotherms to ±0.1–0.3 Å^2^.

#### Brewster Angle Microscopy

The morphology of monolayers was visualized using the Brewster angle microscope (ultraBAM, Accurion GmbH, Goettingen, Germany) with a 50 mW laser-emitting p-polarized light of 658 nm wavelength direct to the air*/*water interface at the Brewster angle (53.2°), 10 × magnification objective, polarizer, analyzer, and a CCD camera. The spatial resolution of the image was 2 μm. The microscope was installed over the KSV 2000 Langmuir trough of total area of 700 cm^2^ (Helsinki, Finland), placed on an antivibration table with an active vibration isolation system.

### Statistical Analysis

Data were presented as mean ± SE. The experiments were repeated at least three times, and each experiment included at least triplicate treatments. The data from the different treatments were statistically analyzed using the SAS ANOVA procedure and the comparisons of the means were performed using Duncan’s Multiple Range test, with PC SAS 8.0. Differences with *P* values less than 0.05 were considered as significant.

## Results

Plasmalemma of calli cultured on the media containing ZEA is richer in galactolipid fraction, especially DGDG, in comparison to control (Table 1). Addition of selenate changes the proportion of the lipid fraction towards a higher content of phospholipids. The main phospholipid in PL fraction is phosphatidylcholine (about 33–35% in all calli). Among others, phosphatidylethanolamine (about 29%), phosphatidylglycerol (about 12%), phosphatidic acid (about 10%), phosphatidylinositol (about 8%), and phosphatidylserine (about 6%) were registered.

**Table 1:** Content of lipid fractions (MGDG, DGDG, and PL) calculated as the percentage of total amount of polar lipids and fatty lipid composition in those lipid fraction (as mol%) in the cells of wheat calli cultured in control conditions (Murashige–Skoog, MS, media) and in MS media supplemented with 30 µM ZEA and 30 µM ZEA + 15 µM Na_2_SeO_4_^2−^

Object	Lipid fraction (mol% of polar lipids)	Fatty acids (mol%)							
		16:0	16:1	18:0	18:1	18:2	18:3	U/S	(18:3/18.2)
*MGDG*									
Control	30.03 ± 0.02^b^	26.3 ± 0.2^c^	tr	9.5 ± 0.3^b^	15.4 ± 0.2^a^	30.1 ± 0.4^a^	18.7 ± 0.2^b^	1.79 ± 0.02^a^	0.621 ± 0.004^b^
ZEA	30.70 ± 0.05^b^	28.0 ± 0.2^a^	–	10.3 ± 0.1^a^	11.8 ± 0.2^c^	30.3 ± 0.3^a^	19.6 ± 0.1^a^	1.61 ± 0.03^c^	0.647 ± 0.005^a^
ZEA + SeO_4_^2−^	28.61 ± 0.05^b^	27.0 ± 0.4^b^	tr	9.4 ± 0.2^b^	14.6 ± 0.4^b^	30.2 ± 0.4^a^	18.8 ± 0.1^b^	1.74 ± 0.02^b^	0.622 ± 0.002^b^
*DGDG*									
Control	25.17 ± 0.04^c^	26.5 ± 0.2^c^	1.0 ± 0.2^a^	11.3 ± 0.1^b^	17.0 ± 0.1^a^	28.5 ± 0.3^a^	15.7 ± 0.2^b^	1.64 ± 0.01^a^	0.551 ± 0.003^b^
ZEA	27.31 ± 0.03^c^	27.9 ± 0.2^a^	–	12.1 ± 0.2^a^	15.1 ± 0.2^b^	28.2 ± 0.3^a^	16.7 ± 0.1^a^	1.50 ± 0.02^b^	0.592 ± 0.004^a^
ZEA + SeO_4_^2−^	24.80 ± 0.04^c^	27.0 ± 0.3^b^	0.8 ± 0.2^a^	11.5 ± 0.1^b^	17.0 ± 0.2^a^	28.2 ± 0.2^a^	15.5 ± 0.2^b^	1.60 ± 0.03^a^	0.547 ± 0.004^b^
*PL*									
Control	44.80 ± 0.05^a^	13.8 ± 0.2^b^	–	2.4 ± 0.1^b^	26.8 ± 0.2^b^	35.8 ± 0.3^a^	21.2 ± 0.2^b^	5.17 ± 0.02^a^	0.592 ± 0.002^b^
ZEA	41.99 ± 0.03^a^	15.3 ± 0.2^a^	–	3.1 ± 0.1^a^	23.7 ± 0.3^c^	35.4 ± 0.2^a^	22.5 ± 0.3^a^	4.43 ± 0.04^b^	0.636 ± 0.003^a^
ZEA + SeO_4_^2−^	46.59 ± 0.04^b^	13.9 ± 0.1^b^	–	2.5 ± 0.1^b^	27.9 ± 0.3^a^	35.4 ± 0.2^a^	20.9 ± 0.2^b^	5.10 ± 0.01^b^	0.590 ± 0.003^b^

U/S was expressed as the sum of unsaturated/saturated fatty acids. Values represent the average ± SE (*n* = 45)

Significant differences (*p* ≤ 0.05) between treatments for each lipid fraction are marked by different letters

The studied lipid fractions contain palmitic (16:0) and stearic (18:0) acids as the main saturated fatty acids, and oleic (18:1), linoleic (18:2), and linolenic (18:3) acids as predominant unsaturated fatty acids (Table 1). In all fractions, traces of more unsaturated fatty acids than 18:3, were also registered. Moreover, in MGDG and DGDG fractions small amount of 16:1 acid was detected. The degree of unsaturation was calculated as the ratio of molar content of unsaturated to saturated fatty acids (U/S). Generally, the plasmalemma of calli cultured on ZEA media shows lower degree of unsaturation than those grown on control, in all investigated fractions. The presence of selenate increases lipid unsaturation to the levels close to those obtained for control. Since the unsaturated lipids represent greater part (about 60–80%) of the total amount, additional parameter describing the changes in the level of the most unsaturated (18:3/18:2) fatty acids was calculated (Table 1). In all lipids, ZEA application results in the increase of the proportion of 18:3–18:2 acids. However, when SeO_4_^2−^ ions are additionally introduced, this ratio decreases.

**Fig. 2:**
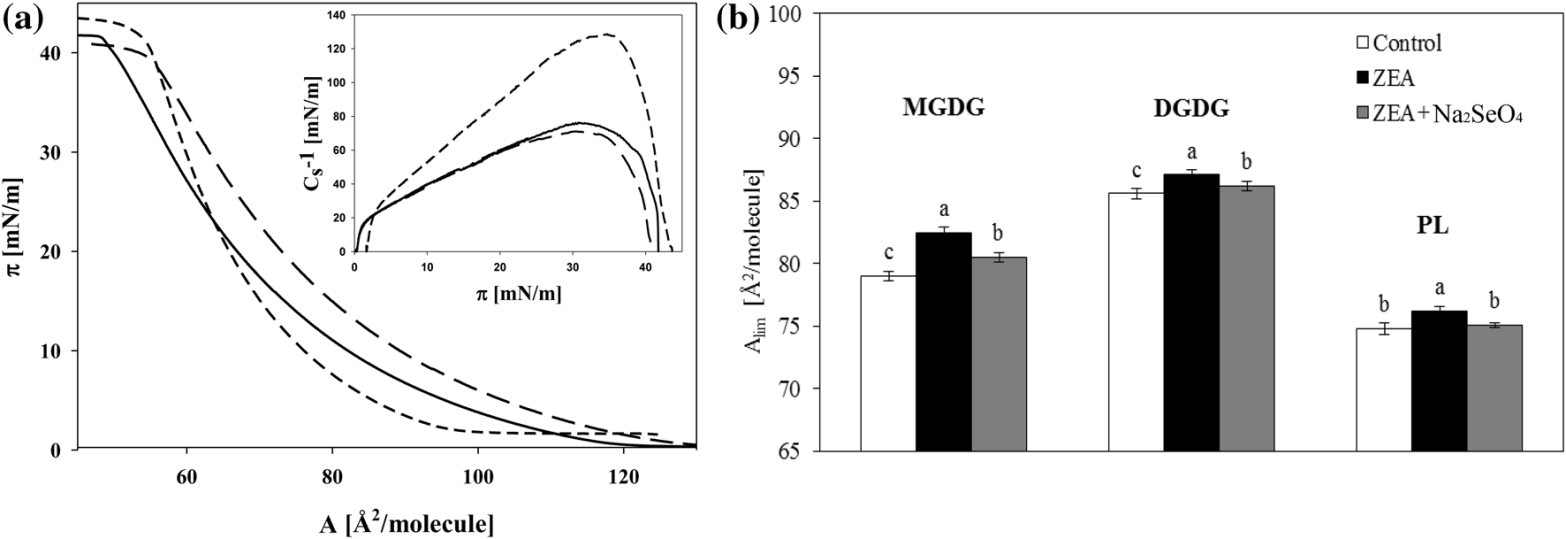
**a**—The surface pressure—area per molecule isotherms recorded for monolayers of lipids extracted from callus grown on control media, spread on water. The fraction of MGDG—*solid line* DGDG fraction—*dashed line* PL fraction—*short dash line*. *Inset* compressibility modulus (*C*_s_^−1^) versus surface pressure (*π*). **b**—Effect of ZEA and selenate ions on the limiting area for lipids extracted from calli cultured for 10 days on MS media (control) and MS media with 30 μM ZEA and with ZEA (30 μM) + SeO_4_^2−^ (15 μM). Values represent the average ± SE (*n* = 5). *Different letters* indicate significant differences between treatments (*p* ≤ 0.05)

The effect of ZEA (and ZEA + SeO_4_^2−^) on the structural properties of plasmalemma of wheat cells was determined on the basis of physicochemical properties of monolayers, prepared from lipids isolated from calli cultured at studied conditions. Figure 2a presents examples of isotherms obtained for PL, DGDG, and MGDG extracted from calli grown on control media. The shapes of isotherms for MGDG and DGDG are similar, but DGDG isotherm is shifted in the direction of higher values of area per molecule. Isotherm for PL has a steeper slope than those recorded for galactolipids. Values of compressibility modulus *C*_s_^−1^ calculated for MGDG and DGDG are also comparable and are about twice smaller than that for PL monolayers.

On the basis of all recorded isotherms, the limiting area (*A*_lim_) was determined. In Fig. 2b the values of *A*_lim_ for all lipid fractions are presented. The largest limiting area is found for DGDG and the smallest one for the PL fraction. However, the differences in the limiting area values between PL and MGDG are small (about 4–5 Å^2^), whereas when PL and DGDG are taken into account, these differences grow to about 15 Å^2^. Zearalenone presence in the culture media of calli results in a shift of isotherms towards higher *A*_lim_ values (Fig. 2b).

**Fig. 3:**
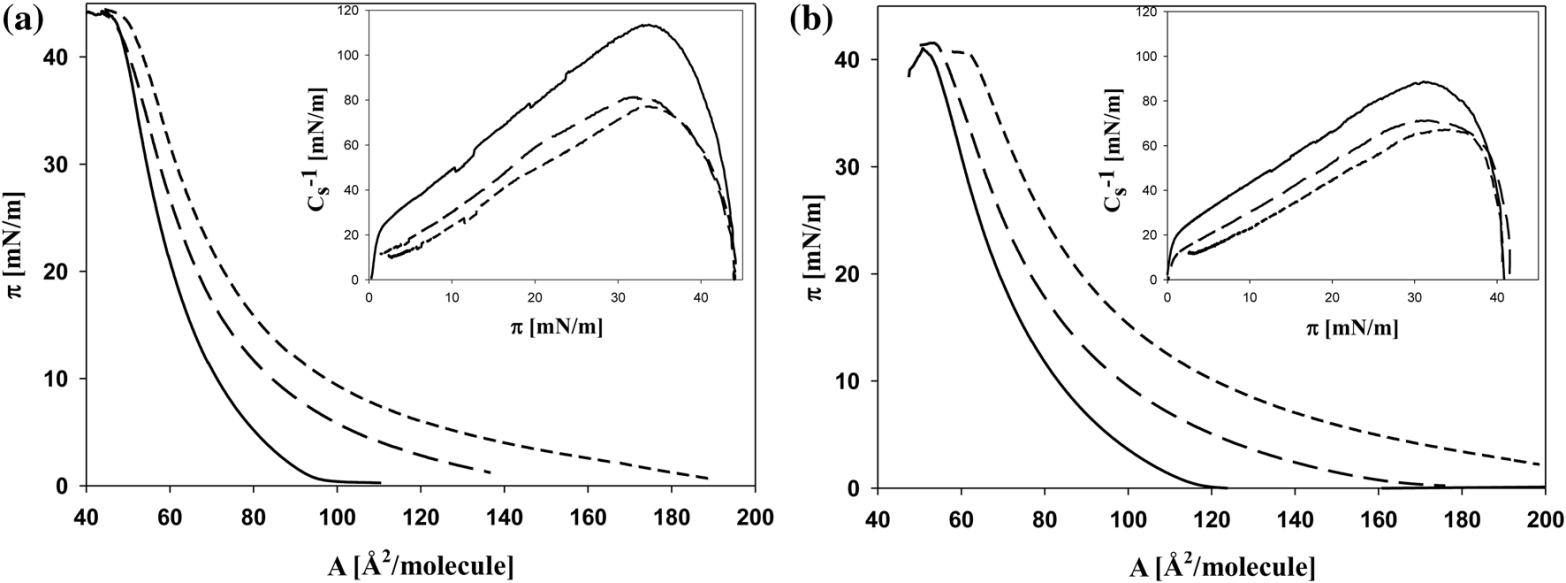
The representative example of surface pressure—area per molecule isotherms for monolayers of PL (**a**) and DGDG (**b**) fractions obtained from plasmalemma of calli cultured on control media, spread on water (*solid line*). The effect of ZEA added into the subphase represents *long dash line* (1.5 × 10^−5^ M) and *short dash line* (3.0 × 10^−5^ M), respectively. *Inset C*_s_^−1^ versus *π*

**Fig. 4:**
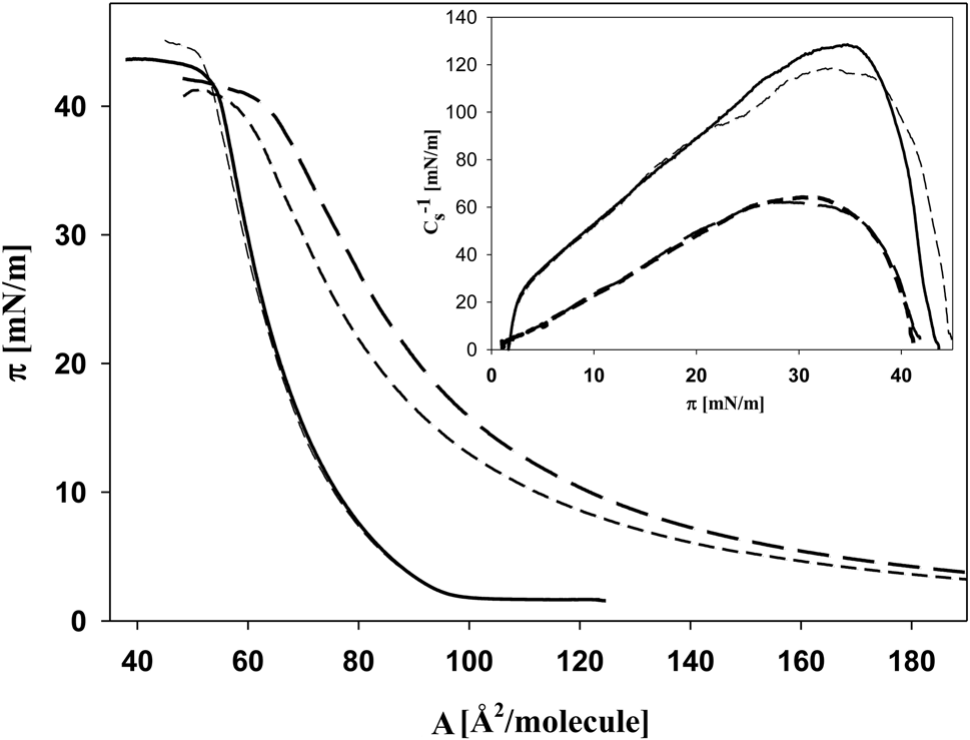
The representative surface pressure—area per molecule isotherms for PL fractions obtained from plasmalemma of calli cultured on control media, spread on water (*solid line*). The effect of ZEA added into the subphase (3 × 10^−5^ M)—*dash line* ZEA + SeO_4_^2−^ (3 × 10^−5^ M + 1.5 × 10^−5^ M)—*thick short dash line* and SeO_4_^2−^ (1.5 × 10^−5^ M)—*short dash line*. *Inset C*_s_^−1^ versus *π*

Described changes in the properties of lipid monolayers are related to the long-term effects of the investigated substances acting during 7 days on cell membranes. The used technique allows also determining the effects of the direct interaction between lipids and ZEA (and ZEA + SeO_4_^2−^) present in the solution, associated with the adsorption of these substances on the membrane surface. In Fig. 3a, b the influence of ZEA (at concentrations 15 and 30 µM) on monolayers formed by PL and DGDG fractions, obtained from the plasmalemma of plants cultured on control media, is shown. These fractions were chosen as those for which the smallest (PL) and the highest (DGDG) values of physicochemical properties were observed during 7 days culture. Introducing of ZEA at 15 µM concentration into a water subphase results in an increase of *A*_lim_ and a decrease of maximum value of *C*_s_^−1^ calculated for layers of both investigated lipid fractions. At higher ZEA concentration (30 µM), a further rise in the *A*_lim_ value (in comparison to control) is observed (about 17.51 and 24.48% increase for PL and DGDG, respectively). The *C*_s_^−1^ parameter shows a downward trend for both PL and DGDG, with a decrease of about 40 mN/m and 23 mN/m, respectively, which accounts approx. 33.6 and 25% of the values calculated for control. The simulation of the influence of both ZEA and SeO_4_^2−^ on the monolayers of calli cultured in control conditions are investigated on the example of PL fraction (Fig. 4). The presence of SeO_4_^2−^ ions in a subphase does not influence the surface parameters of PL monolayers, whereas ZEA alone leads to an increase of *A*_lim_ by about 42% and to a decrease of the maximum value of *C*_s_^−1^ by about 49% (relative to control). In the case of the addition of ZEA + SeO_4_^2−^ mixture to the subphase, the isotherm of lipids on ZEA is shifted towards the isotherm recorded for control. Consequently, the *A*_lim_ decreases (as compared to values for lipid monolayer on ZEA). The maximum value of *C*_s_^−1^ is similar to that found when only ZEA is applied.

**Fig. 5:**
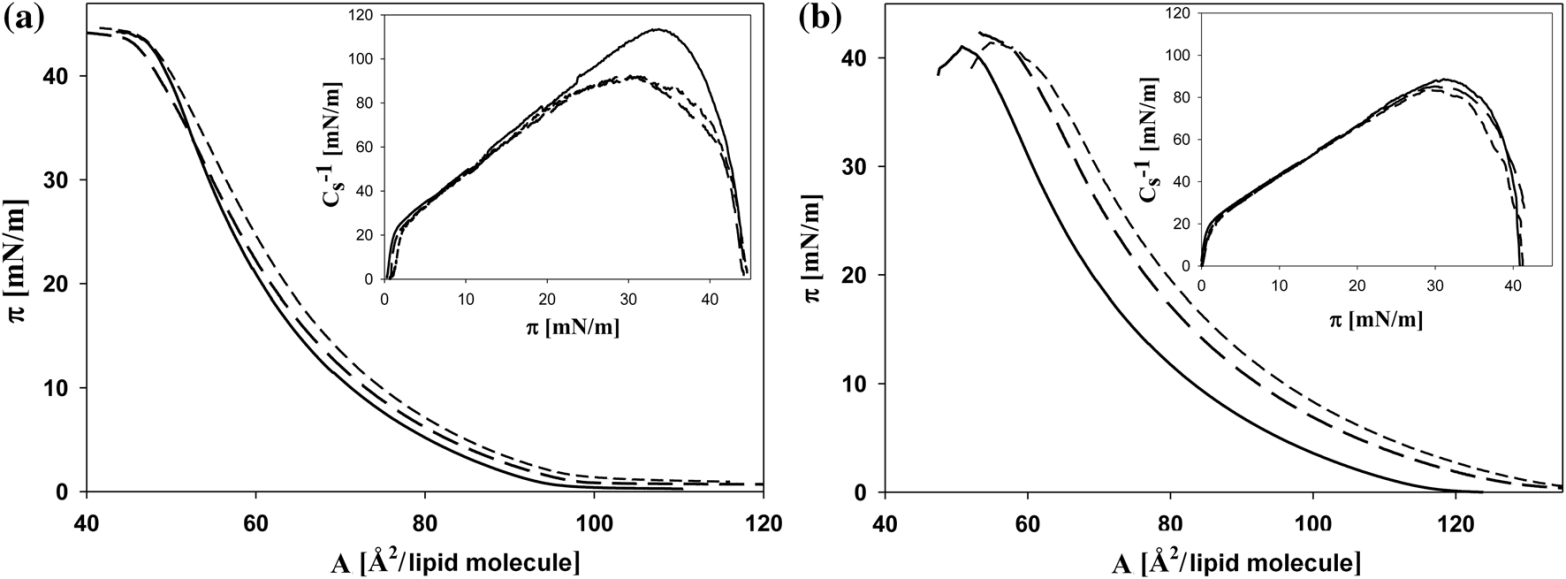
The representative example of surface pressure—area per lipid molecule isotherms for PL (**a**) and DGDG (**b**) fractions obtained from plasmalemma of calli cultured on control media (*solid line*) and for mixtures of these lipids with ZEA at 2:1 (mol:mol)—*dash line* and 1:1 (mol:mol)—*short dash line*, spread on water. *Inset C*_s_^−1^ versus *π*

In addition, the influence of ZEA on the surface properties of layers was studied when the layers were formed by the mixtures of ZEA + PL (and +DGDG). ZEA, as a substance showing both hydrophilic and hydrophobic properties, can be dissolved in chloroform and together with lipid can build monolayer on water subphase (Fig. 5a, b). It is easily seen, that the ZEA mixed with lipids causes a greater shift (towards higher molecular areas) of isotherms of DGDG monolayer (Fig. 5b) than that of PL (Fig. 5a). However, when *C*_s_^−1^ is taken into account, a larger decrease is seen in the case of PL (the insets of Fig. 5a, b). Therefore, ZEA modifies the compressibility of PL monolayer to a greater extent.

**Fig. 6:**
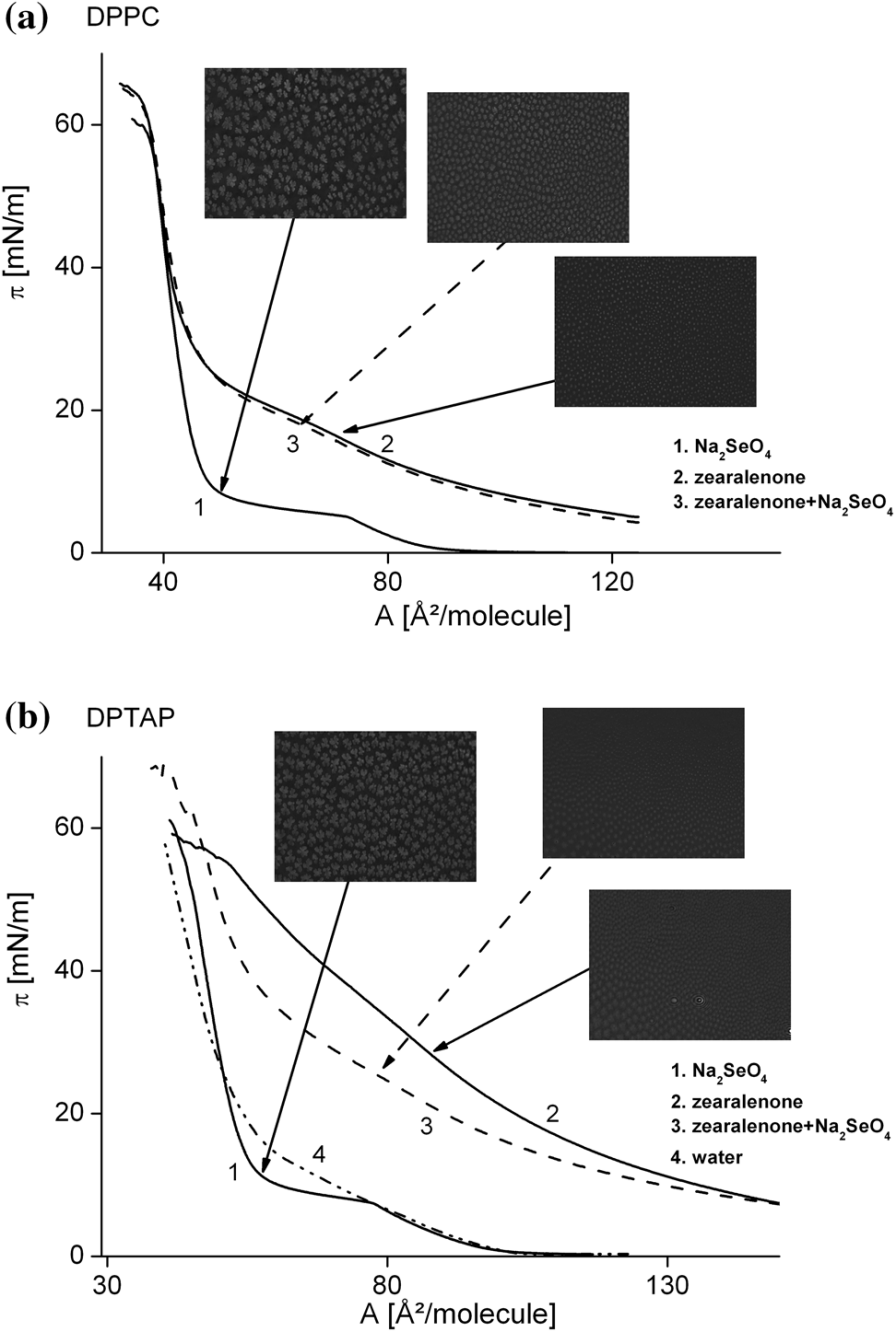
The surface pressure—area per molecule isotherms and BAM images of DPPC (**a**) and DPTAP (**b**) monolayers on three different subphases: ZEA (10^−5^ M), SeO_4_^2−^ (1.5×10^−5^ M), and ZEA + SeO_4_^2−^ (10^−5^ M + 1.5×10^−5^ M). The isotherm of DPTAP monolayer on pure water is added for comparison (**b**). The *arrows* indicate the compression stages at which BAM images were recorded

In Fig. 6, the *π–A* isotherms of DPPC (A) and DPTAP (B) monolayers on three different subphases (ZEA, SeO_4_^2−^, and ZEA + SeO_4_^2−^) are presented with BAM images. In the case of DPPC on the water solution of selenate ions (Fig. 6a), the isotherm shape is the same as that observed for DPPC monolayer on pure water, i.e., the plateau corresponding to the LE (liquid expanded)—LC (liquid condensed) coexistence is clearly visible. This is not surprising, considering the fact that the divalent anions do not interact with the DPPC monolayer (Gzyl-Malcher et al. 2011). In BAM image, the LC phase appears as bright structures surrounded by a dark LE phase. These LC domains have multilobed structure. The domain shape is unusual but has been observed before (Chen et al. 2010; Telesford et al. 2015; Miñones et al. 2002). When ZEA is injected to the subphase, the DPPC monolayer is expanded and the LE/LC coexistence region is shifted towards higher surface pressures. This means, that the DPPC monolayer must be compressed to higher pressures to undergo the LC/LE transition. The domains recorded for DPPC monolayer on ZEA solution are smaller and have less branched structure. Due to the hydrophobic properties of ZEA, it can penetrate into monolayer. With both solutes (SeO_4_^2−^ ions and ZEA) in the subphase, the isotherm shape is almost unchanged compared to that recorded for DPPC monolayer on ZEA. However, the BAM image reveals marked differences. The domains have a shape and a size intermediate with respect to those observed for DPPC monolayer on selenate ions or zearalenone alone. Therefore, although the selenate anions alone do not influence DPPC monolayer (Gzyl-Malcher et al., 2011), they can protect the lipid domains from the impact of ZEA. On the contrary, it was shown in (Gzyl-Malcher et al., 2011) that SeO_4_^2−^ ions have a great impact on DPTAP monolayer, causing its condensation (Fig. 6b). In the *π–A* isotherm of DPTAP on Na_2_SeO_4_ solution, one can distinguish a clear phase transition between LE and LC phases, similar to that in DPPC isotherm. In the BAM image, there are visibly large, well-developed domains. When ZEA instead of SeO_4_^2−^ ions is present in the subphase, the DPTAP monolayer is expanded. This is shown by the isotherm shape—the LE/LC phase transition is shifted towards higher surface pressures and it is not as pronounced as in the presence of SeO_4_^2−^ anions. The monolayer expansion is also evidenced by the shift of the isotherm to higher molecular areas and by the decrease in its slope in the LC region. The domains observed in the BAM image are much smaller and they appear at higher surface pressures compared to monolayer spread on water solution of selenate. When both solutes (ZEA and SeO_4_^2−^) are present in the subphase, the *π–A* isotherm is again shifted towards lower molecular areas and its slope is steeper. However, the BAM image is the same as that with ZEA alone. Therefore, despite the condensing effect of SeO_4_^2−^ anions, it is ZEA present in the subphase that has a decisive influence on the domain structure of DPTAP monolayer.

**Fig. 7:**
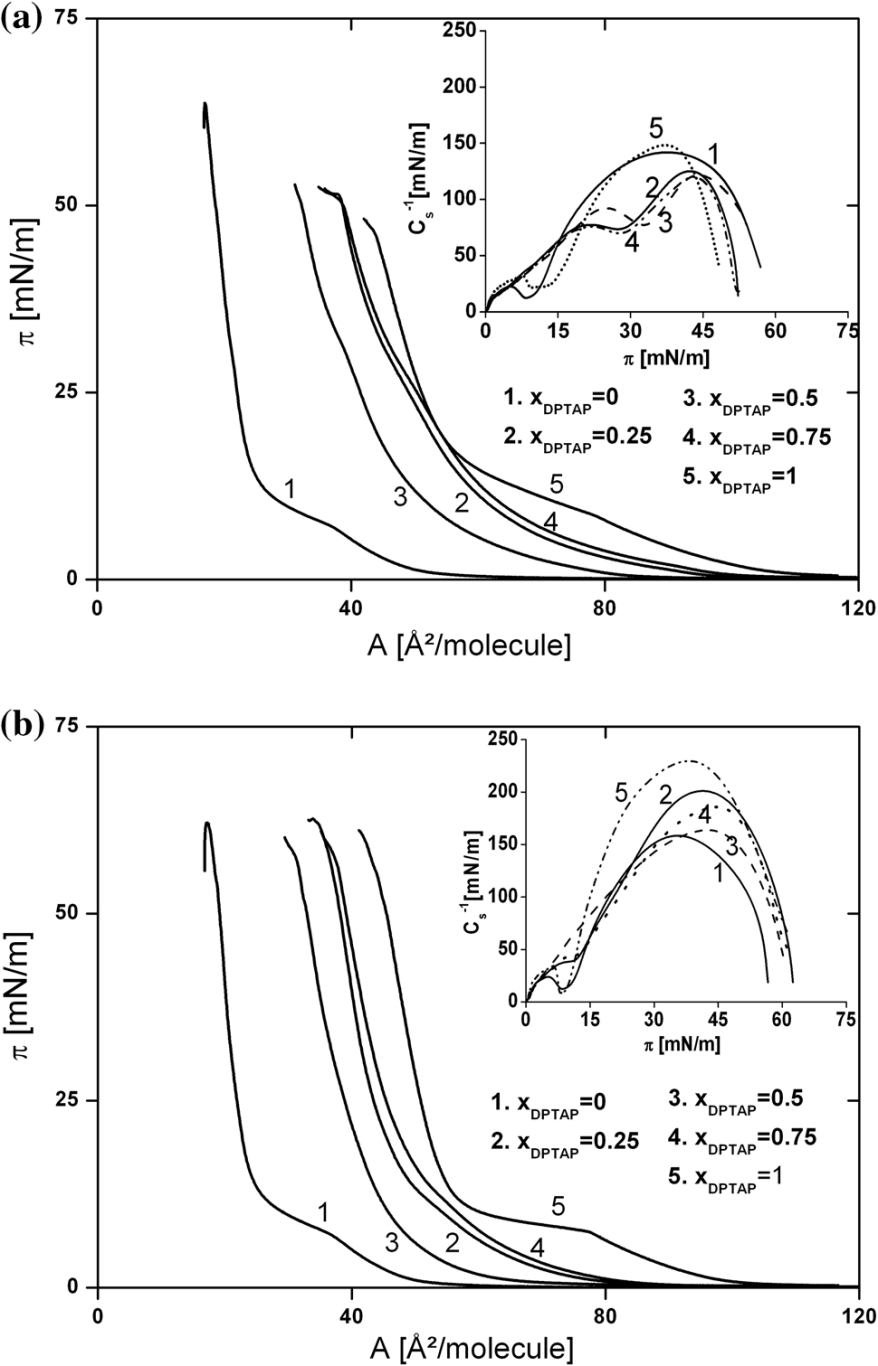
The surface pressure—area per molecule isotherms recorded for DPPC:ZEA (1:1; mol:mol)/DPTAP mixed monolayers with various contents of DPTAP, spread on water (**a**) and on the water solution of selenate (**b**). *Inset C*_s_^−1^ versus *π*

In Fig. 7, the *π–A* isotherms recorded for DPPC:ZEA/DPTAP mixed monolayers with various contents of DPTAP, spread on water (A) and on water solution of SeO_4_^2−^ ions (B), are presented. The most striking feature is that the isotherms are not arranged according to the increasing molar fraction, i.e., the isotherm corresponding to the monolayer with *x*_DPTAP_ = 0.25 is shifted to larger molecular areas and is located closer to the isotherm of pure DPTAP monolayer than the isotherm recorded for the mixture with *x*_DPTAP_ = 0.5. This suggests the occurrence of more repulsive interactions between components in a mixed monolayer of this composition. By comparing Fig. 7a, b, it is easily seen that the part of the isotherms corresponding to the liquid-condensed state are less steep when recorded for mixed monolayers on pure water. In addition, the LC/LE phase transition occurs at higher surface pressures in comparison to monolayers spread on SeO_4_^2−^ solution. Thus, the monolayers spread on water are more expanded. To validate this suggestion the compressibility modulus values were calculated and plotted as a function of surface pressure (the inset of Fig. 7). When SeO_4_^2−^ ions are present in the subphase, the *C*_s_^−1^ values are much higher and the minimum value corresponding to the phase transition is shifted to lower surface pressures. Therefore, the condensing effect of SeO_4_^2−^ ions, observed above in the BAM experiments, is found also in this study. The only exception is an equimolar mixture of DPPC and ZEA, for which no change in the shape and the position of the isotherm is found, as a result of the aforementioned absence of interaction between this zwitterionic lipid and divalent anions. Additionally, for all mixed systems, increased monolayer stability against the collapse process is found in the presence of SeO_4_^2−^ ions.

To determine the interactions between components in mixed monolayer, the excess free energy of mixing ΔG^EXC^ was calculated, according to (Birdi 1989):

$$\Delta G^{\text{EXC}} = N_{A} \int\limits_{0}^{{\pi_{2} }} {\left( {A_{12} - x_{1} A_{1} - x_{2} A_{1} } \right)} \, d\pi,$$

where *N*_*A*_ is the Avogadro’s number, *A*_1_, *A*_2_, and *A*_12_ are the mean molecular areas of the pure components (1 or 2) and their mixture (12), and *x*_1_ and *x*_2_ are the mole fractions of the components in the mixture.

**Fig. 8:**
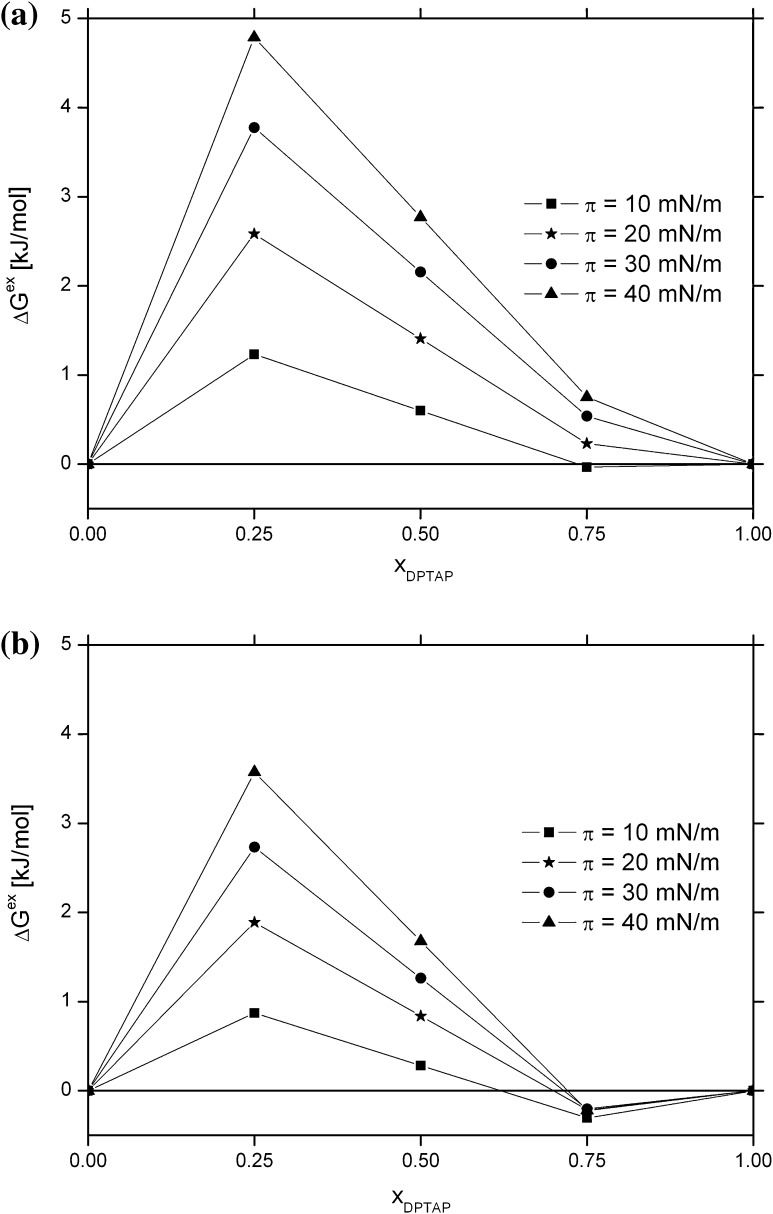
The variation of excess free energy of mixing (ΔG^EXC^) versus molar fractions of DPTAP (x_DPTAP_) for DPPC:ZEA (1:1; mol:mol)/DPTAP monolayer, spread on water (**a**) and on the water solution of selenate (**b**), at four surface pressures: 10, 20, 30, and 40 mN/m

The variation of Δ*G* ^EXC^ for DPPC:ZEA/DPTAP monolayer against molar fractions of DPTAP at four surface pressures (10, 20, 30, and 40 mN/m) is shown in Fig. 8. Generally, the positive values of Δ*G* ^EXC^ are obtained for all mixed monolayers, with the exception of the one with *x*_DPTAP_ = 0.75, spread on SeO_4_^2−^ solution. Furthermore, the amount of Δ*G* ^EXC^ increases with the increasing surface pressure. This is a result of the reduced distance between the molecules when the monolayer is compressed, whereby the repulsive interactions between them grow.

## Discussion

The main reason for the undertaken experiments is to find the relationship between zearalenone present at stressogenic concentration and selenate ions as a result of their impact on lipid monolayers, to elucidate the possibility of the protective effects of selenate against the toxicity of ZEA in biomembranes. The generally accepted indicator of stress intensity is lipid peroxidation (measured as an increase of malonyldialdehyde concentration) which informs about the increase of fatty acid saturation by reactive oxygen species (Ayala et al. 2014). Excessive production of reactive species, produced under various environmental stresses, leads to progressive oxidative damage and ultimately cell death, as consequences of the oxidative stress (Sharma et al. 2012). The influence of ZEA on the induction of oxidative stress in the studied plant cells is confirmed by an increase of fatty acid saturation of investigated lipid fractions. Changes in fatty acid proportions under different stresses were observed as both increase (Zhang et al. 2005) and decrease (Allakhverdiev et al. 2001) of saturation, and were interpreted not only as the effect of reaction of reactive oxygen species with unsaturated bonds present in the lipid molecules, but also as an important mechanism of cell adaptation under changed environmental conditions. An increase of biomembrane saturation and the associated greater stiffness is considered to be a protective mechanism preventing exogenic molecules from penetrating into cells via mechanical blocking of spontaneous diffusion and/or membrane transporters (Kumar 2014).

The determined effect of ZEA on the quantitative composition of the main lipid fractions of wheat plasmalemma indicates that such stress changes the cell metabolism to produce larger amount of less polar galactolipids in comparison to negatively charged phospholipids. The presence of SeO_4_^2−^ ions restores the proportions that are characteristic of the control plant. Similar changes in lipid composition were observed in plastid envelope membranes during Cd-stressed wheat calli (Filek et al. 2009). An increase of galactolipid/phospholipid proportion in the cells cultured on media supplemented with ZEA and a decrease of this ratio when SeO_4_^2−^ ions are additionally introduced, observed in the present experiments, confirm the significance of lipid composition in the mechanism of cell protection in stress conditions initiated by toxin.

The determined physicochemical parameters of monolayers built from lipids extracted from calli allow indicating that 7-day contact of biomembranes with ZEA reduces the maximal possible packing density of molecules in membranes (the increase in *A*_lim_), whereas SeO_4_^2−^ ions present together with ZEA partially prevent loosening of lipid packing. The packing density of membranes is the resultant effect of the interaction between both polar and hydrophobic groups within lipid layer. Thus, the largest *A*_lim_ is obtained for DGDG fraction with the largest polar group and the smallest *A*_lim_ for PL fraction. Considering an increase of fatty acid peroxidation (U/S ratio) in cells cultured under ZEA stress conditions, an increase of membrane condensation (decrease of *A*_lim_) can be expected. The observed opposite effect, for all the studied lipid fractions, is probably connected with the favorable synthesis of the most unsaturated (18:3) lipid in calli cultured on media with ZEA. The analysis of *π*−*A* isotherms obtained for the monolayers of extracted lipids allows to evaluate the arrangement of lipid molecules in monolayers (Wnętrzak et al. 2013). The decreased stiffness and the formation of more expanded monolayers are characteristic for all the lipid fractions obtained from calli cultured for 7 days in ZEA presence. The formation of liquid-type films was proved by the values of compressibility modulus *C*_s_^−1^. By comparing the parameters characterizing the studied lipid layers, it is evident that lipids obtained from cells cultured in the presence of ZEA form less condensed membranes, with stronger loosening effect in the case of galactolipid than phospholipid fractions. For lipids isolated from the plasmalemma of calli cultured on media with additional supplementation of SeO_4_^2−^ ions, the determined parameters characterizing the monolayers have values close to that obtained for monolayers of control, indicating the significant effect of SeO_4_^2−^ ions on ZEA-modified structural parameters of membranes.

It seems that influence of SeO_4_^2−^ ions is especially important in the initial stage of interaction of ZEA with membranes (Fig. 3). Regarding the isotherms for both galacto- and phospholipids of control calli spread on the subphase that contained ZEA, obtained surface parameters indicate that the direct interaction of this toxin with lipids reduces the degree of packing of lipid molecules in the monolayer. Such effect can be considered as a possible result of ZEA penetrating a lipid layer. Such explanation may be supported by the fact that similar changes of surface parameters were obtained for two-component monolayers prepared from mixed lipid + ZEA in chloroform solutions. The possibility of incorporation of ZEA into lipid monolayers was indicated also by Gzyl-Malcher et al. (Gzyl-Malcher et al. 2007). The higher effect of ZEA on structural parameters of phospholipids, in comparison to galactolipids, in the initial stage of action (when ZEA is directly applied to the subphase), may be the reason why remodeling of lipid composition in the direction of galactolipids, as less penetrated by ZEA, is observed during prolonged (7 days) contact of cells with the toxin. It is also indicated that SeO_4_^2−^ presence, in the same concentration as was used in culture media, does not influence *π*−*A* isotherms, whereas SeO_4_^2−^ added together with ZEA, partly reduces the effect of this toxin on the surface properties of lipid layers.

The experiments in which the effect of ZEA on monolayers formed by selected lipids, DPPC and DPTAP, was examined confirm the penetration by this toxin of both zwitterionic and cationic monolayers. The expanded membranes in ZEA presence may be more permeable for substances which are unfavorable for cell’s proper function and may partly explain the destructive action of ZEA. The SeO_4_^2−^ ions present in the subphase affect the LC domains of DPPC modified by ZEA in higher degree than when positively charged structures occur in the membrane. Thus, the polarity of membranes is an important factor in both ZEA toxicity and SeO_4_^2−^ protection of biomembranes. The positive values of Δ*G* ^EXC^ indicate that the interactions between components in mixed monolayer are more repulsive (or less attractive) than interactions between the same molecules in one-component monolayer. In (Gzyl-Malcher et al., 2011) attractive interactions between DPPC and DPTAP were observed. Thus, the presence of ZEA in the monolayer negatively affects the molecular arrangement at the water/air interface. This effect is greatest for the mixture containing the largest amount of ZEA. When the content of ZEA decreases, the repulsive interactions between monolayer components also decrease. Introduction of selenate ions to the subphase also leads to reduction in the value of Δ*G* ^EXC^ (Fig. 8b). Moreover, for mixed monolayer with the highest content of DPTAP, negative Δ*G* ^EXC^ values are calculated. This can be explained by the neutralization of positive net charge of this monolayer by selenate anions present in the subphase. This in turn leads to a reduction in the repulsive interactions, allowing a closer packing of the molecules.

## Conclusions

On the basis of the research it can be stated that zearalenone can localize in the lipid monolayers, and the degree of penetration depends on their polarity. Zearalenone may affect the lipid monolayer both by hydrogen bonds, in which hydroxyl, ketone, and lactone groups of ZEA are involved, as well as by van der Waals interactions. Because galactolipids are more susceptible to the influence of zearalenone, remodeling of the composition of the membrane in the direction of a higher content of phospholipids seems to be an important stage of a cell defense mechanism counteracting toxic effects of this toxin. It was also shown that the presence of selenate anions in the water subphase reduces the penetration of zearalenone to the monolayer. In case of monolayers containing positively charged groups, the electrostatic attraction between them and the selenate anions may hinder access of zearalenone molecules to the monolayer. The way in which selenate ions reduce the interaction of ZEA with monolayers of neutral lipids, however, is a question that will require further research. The possible cause of the weakened influence of ZEA might be the interaction with selenate anions through H-bonding.
